# *Veillonella parvula* promotes the proliferation of lung adenocarcinoma through the nucleotide oligomerization domain 2/cellular communication network factor 4/nuclear factor kappa B pathway

**DOI:** 10.1007/s12672-023-00748-6

**Published:** 2023-07-14

**Authors:** Wen Zeng, Yuhuan Wang, Zhe Wang, Mengge Yu, Kang Liu, Chengzhu Zhao, Yiyun Pan, Shudong Ma

**Affiliations:** 1 Oncology Research Institute, Ganzhou Cancer Hospital, Gannan Medical University, Huayuan Road No.19, Shuidong Town, Zhanggong District, Ganzhou, 341000 Jiangxi Province China; 2 Department of Oncology, Nanfang Hospital, Southern Medical University, 1838 North Guangzhou Avenue, Baiyun District, Guangzhou, 510000 Guangdong Province China; 3 The First Clinical College, Gannan Medical University, Ganzhou, Jiangxi Province China

**Keywords:** *Veillonella parvula*, Lung adenocarcinoma, 16 s rRNA sequencing, LLC cells, CCN4 expression

## Abstract

Enrichment of *Veillonella parvula* in the lung microbiota is strongly associated with non-small cell lung cancer (NSCLC) and induces the progression of lung adenocarcinoma in vivo, but its actual role and mechanism remain unexplored. This study analyzed the correlation between NSCLC and *V. parvula* abundance based on 16 s rRNA sequencing results. The effects of *V. parvula* on the progression of lung adenocarcinoma were observed in vivo and in vitro using a C57 bl/6j mouse tumor-bearing model, a bacterial cell co-culture model, combined with transcriptome sequencing, and a TCGA database to explore and validate the growth promotion of lung adenocarcinoma by *V. parvula* and its molecular mechanism. 16 s rRNA sequencing revealed that *V. parvula* was significantly enriched in lung adenocarcinoma. In vivo, *V. parvula* promoted the growth of lung adenocarcinoma in mice by suppressing the infiltration of tumor-associated T lymphocytes and peripheral T lymphocytes. It showed a higher affinity for lung adenocarcinoma in vitro and promoted lung adenocarcinoma cell proliferation through adhesion or intracellular invasion. Further analysis of differential gene expression and KEGG enrichment by transcriptome sequencing revealed that *V. parvula* induced CCN4 expression and activated NOD-like receptor and NF-κB signaling pathway in lung adenocarcinoma cells. Further analysis clarified that *V. parvula* promoted activation of the NF-κB pathway via Nod2/CCN4 signaling, which promoted lung adenocarcinoma cell proliferation. Thus, *V. parvula* mediates activation of the Nod2/CCN4/NF-κB signaling pathway to promote non-small cell lung adenocarcinoma progression, thereby providing a potential target for diagnosing and treating lung adenocarcinoma.

**Graphical Abstract:**

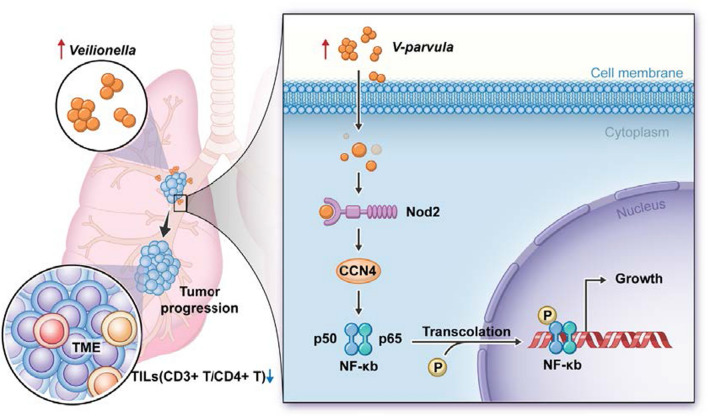

**Supplementary Information:**

The online version contains supplementary material available at 10.1007/s12672-023-00748-6.

## Introduction

Lung cancer has long been the most prevalent cancer globally, accounting for 18.4% of all cancer-related deaths (1, 2). Smoking is a major causative factor in the pathogenesis of lung cancer. However, only 15% of lung cancer episodes are associated with smoking, suggesting that vital environmental factors other than smoking contribute to the development of lung adenocarcinoma (LUAD) (3). Infectious diseases are the third primary etiology of cancer worldwide, accounting for up to 16.1% of cancers associated with viruses, bacteria, and other pathogenic microorganisms (4, 5). The role of bacteria in cancer has received increasing attention with the advancement in microbiota research. A significant update to the Hallmarks of Cancer in 2021 defines “Polymorphic microbiomes” as novel important markers with the ability to drive tumor progression and acquire other cancer markers (6).

Research shows that dysbiosis is an important factor in the development and progression of colon and breast cancers; microbiota also plays a key role in lung cancer (7, 8). Therefore, it is important to investigate the role and mechanism of flora and specific genera in lung cancer for its diagnosis and treatment. The lung microbiota in healthy adults mainly comprises Bacteroidetes and Firmicutes (9, 10). Studies have reported that the lung microbiome is associated with the development of lung diseases (11, 12). Recently, there has been growing interest in exploring the relationship between the lung microbiome and lung cancer. However, most studies have not yet investigated the impact of the lung microbiota on the pathogenesis of lung cancer. The removal of lung commensal bacteria from mice has been shown to suppress lung cancer; in contrast, transplantation of lung commensal bacteria from mice with advanced lung cancer promoted inflammation and progression of lung cancer (13). Yan et al. reported that the levels of Capnocytophaga and Veillonella were significantly elevated in the salivary microbiome of lung cancer patients, suggesting that the salivary microbiome may be a potential biomarker for lung cancer (14). Several studies have reported that the lung microbiome is involved in the pathogenesis of lung cancer (15–17). One possible mechanism is that bacteria induce chronic inflammation by promoting the proliferation of airway epithelial cells by producing pro-inflammatory factors, ultimately leading to cell transformation and tumor formation (18). Although the association between the lung microbiome and lung cancer has been reported, the underlying mechanisms of their interactions have not been fully explored. Understanding these mechanisms may provide insights into the pathogenesis of lung cancer.

A previous study has indicated a close association between *V. parvula* enrichment and lung cancer, highlighting its potential as a predictive biomarker for lung cancer (19). *V. parvula* is a specialized anaerobic gram-negative coccus located primarily in oral mucosa and other ecological niches, such as the lung, intestine, and vagina (20). The enrichment of *V. parvula* in the lower respiratory tract is correlated with stages of lung cancer, and perfusion of *V. parvula* through the airways affects the imbalance of the lung microbiota and modifies the airway transcriptome, thus promoting the progression of spontaneous lung cancer in mice (21, 22). *V. parvula* may be a driver of lung cancer progression, but its more detailed role and molecular mechanisms remain unclear.

In this study, we hypothesize that *V. parvula* can promote the growth of LUAD by direct or indirect mechanisms. The aim of our investigation is to confirm the role of *V. parvula* in promoting LUAD and explore its mechanism of action. This could help in designing targeted therapies and improving the prognosis for patients with LUAD.

## Methods

### 16 s rRNA gene sequencing

Shanghai Majorbio Bio-pharm Technology Co, Ltd performed 16 s rRNA gene sequencing, and the clinical and sequencing data and analysis methods have been described in detail in our previous study (23). All patients provided written informed consent. This study was approved and supervised by the Ethics Committee of the Ganzhou Cancer Hospital (GZ-KLS-No.202105) in accordance with the ethical principles for medical research involving human subjects delineated in the Declaration of Helsinki.

### Bacterial strains and culture conditions

*Veillonella parvula* ATCC®10790TM (#0867) was purchased from ATCC, and *Escherichia coli* MG1655 (700926) was a gift from the Department of Microbiology, School of Public Health, Southern Medical University (Guangzhou, China). *Veillonella parvula* was cultured in tryptic soy agar (TSA) or tryptic soy broth (TSB) containing 5% defibrinated sheep blood in AnaeroPack-Anaero (Mitsubishi Gas Chemical Company, Inc., Japan) under anaerobic conditions at a constant incubator temperature of 37 °C for 48–72 h, while *E. coli* MG1655 was cultured in lysogeny broth at 37 °C on a constant temperature shaker.

### Cell culture

Mouse Lewis lung carcinoma (LLC) cells (CL-0140), human non-small cell LUAD A549 cells (CL-0016) and human bronchial epithelial cell line BEAS-2B were purchased from Procell Life Science & Technology Co., Ltd (Wuhan, China). LLC cells were cultured in Dulbecco’s Modified Eagle’s Medium (DMEM) (high glucose), A549 cells in DMEM-F12 medium, and BEAS-2B cells in RPMI-1640 medium, all containing 10% fetal bovine serum and 1% penicillin–streptomycin solution, in a 5% CO_2_ incubator at 37 °C.

### Bacterial adhesion assay

The bacterial adhesion assay was performed as previously described (24). A549 or LLC cells at a concentration of 1 × 10^5^ cells/well were inoculated in 24-well plates with *V. parvula* or *E. coli* MG1655 at a multiplicity of infection (MOI) of 100 and co-cultured for 1 h under anaerobic conditions. Cells were washed three times with phosphate-buffered saline (PBS); 100 μL of ddH2O containing 0.1% Triton X-100 was then added to the lysate cells for 20 min, followed by 300 μL of TSB. The recovered cell lysate, diluted in a gradient, was inoculated into TSA, and the colony count was calculated after incubation under anaerobic conditions for 48 h. *E. coli* MG1655 was used as a negative control.

### Animal experiments

SPF grade, 4–5-week female C57 bl/6j mice were purchased from Slack Jingda Experimental Animal Co., Ltd. (SCXK [Xiang] 2019–0004) (Hunan, China). All animal experiments were conducted in compliance with the ARRIVE (Animal Research: Reporting of In Vivo Experiments) guidelines (25). The protocols followed the principles of reduction, refinement, and replacement and were approved by the Animal Ethics Committee of Gannan Medical University (Ganzhou, China). Mice (n = 3–5 per plastic cage) were kept under controlled conditions with cycles of 12-h daylight and 12-h darkness. Antibiotic-containing drinking water (0.2 g/L of ampicillin, neomycin, and metronidazole and 0.1 g/L of vancomycin) was given for one week, and then the mice were randomly divided into groups.

Mice were anesthetized by inhalation of 2% isoflurane at 4L/min Fresh gas flow, then suspended by their dorsal incisors upon an elastic cord; a blunt pair of forceps was used to ventrally pull the tongue forward to expose the larynx, and the vocal cords were visualized. Using a gel loading tip, 50 μL suspensions (containing 5 × 10^5^ LLC cells) were deployed into the trachea of the mice. From the third day of inoculation of LLC cells, 50 μL of *V. parvula* (optical density at 600 nm [OD_600_] = 1.0) and PBS were administered in the same manner as intratracheal inoculation. *V. parvula* or PBS was deployed into the trachea of the mice once every three days, during which changes in body weight were recorded. They were monitored, and no mice developed peritonitis. On the 24^th^ day, mice were euthanized with a flow rate of 25% CO_2_ per minute of chamber volume. Cardiac and respiratory arrests were observed for more than 5 min to verify animal death, and no secondary/physical method of euthanasia was used.

Antibiotic pre-treatment of C57 bl/6j for one week was followed by random grouping and conventional construction of the C57 bl/6j model of LLC subcutaneous transplantation tumor. Under aseptic manipulation, approximately 5 mm^3^-sized transplanted tumors were inoculated into the right dorsal flank of the mice. On the third day of inoculation, 50 μL of *V. parvula* (OD_600_ = 1.0) was injected at the inoculation site. An equal volume of heat-inactivated *V. parvula* was used in PBS in the control group and treated once every three days. During this period, the long (L) and short (W) diameters of transplanted tumors were observed and recorded. Subcutaneous tumors exceeding 20 mm in length and diameter constituted an endpoint. Subcutaneous transplanted tumor tissue and spleen tissue were collected on the 28^th^ day, and tumor weight and volume (V) were recorded as V = ½ × L × W × W. Tumor tissues were used for pathological sectioning and transcriptome sequencing, and lymphocytes were isolated from spleen for flow cytometry detection.

### Electron transmission microscopy

Cells (1 × 10^6^) from logarithmic growth phase A549 or LLC cells were inoculated in 6-well plates and co-cultured with *V. parvula* or *E. coli* MG1655 with MOI = 100 for 1 h, fixed overnight in 2.5% glutaraldehyde, and used to prepare samples for electron microscopy. The morphology of *V. parvula* and *E. coli* MG1655 was observed, along with the position of bacteria in relation to cells.

### RNA sequence

Fresh subcutaneous tumor tissue samples were collected, and approximately 100–200 mg was fixed with RNA later solution; total RNA from these samples was extracted with TRIzol reagent (Thermo Fisher Scientific), and RNA sequencing (RNA-seq) was performed at Shanghai Meiji Biological Co (Shanghai, China). DESeq2 was used for differential expression analysis with a screening threshold of |log2FC|> = 1.4 and *p*-value < 0.05. KEGG enrichment analysis of differentially expressed genes was performed using the R language ClusterProfiler package with a screening condition of *p*.adj < 0.05 and q-value < 0.2.

### Construction of RNAi-transfected and stably transfected cell lines

Shanghai Hanheng Biological Co. Ltd. (Shanghai, China) provided the lentiviral vector (pHBLV-U6-MCS-CMV-ZsGreen-PGK-PURO) carrying sh-CCN4 and control, with the targeting sequence 5′-GGCUGUGUGAGUGCUGUAAGAUG-3′. LLC cells (5 × 10^4^) in the logarithmic growth stage were inoculated into 24-well plates (MOI = 100) with lentiviral infection and screened with 1 μg/mL puromycin for a stable sh-CCN4-expressing LLC cell line (Lv-CCN4-RNAi). An empty-vector containing line (Lv-NC) was used as the control.

LLC and A549 cells were inoculated into 24-well plates and transfected with 100 nM si-m-Nod2 (siB101229124752-1–5, RIBOBIO, Guangzhou, China) and 50 nM si–h-Nod2 (siG000064127A-1–5, RIBOBIO, Guangzhou, China), respectively, using RboFECT™CP Reagent and incubated for 72 h before the assay.

### EdU assay

The EdU assay was employed to measure the synthesis of new DNA and identify cell proliferation activity. Transfected cells (3 × 10^5^) in the logarithmic growth phase were inoculated into 6-well plates. The medium was changed after cell walling and co-cultured with *V. parvula* at MOI = 100 under anaerobic conditions for 12 h; the plates were then transferred to a 5% CO_2_ incubator at 37 °C and incubated for 24 h. The culture medium was changed, followed by incubation with 10 μM EdU for 2 h, stained with Alexa Fluor 594 BeyoClick™ EdU Cell Proliferation Kit (Beyotime, C00788L), observed under a fluorescent inverted microscope (Vert. A1; Zeiss, Germany) and photographed.

### Flow cytometry

The flow cytometry technique was performed to quantify CD3 + , CD4 + , and CD8 + T cells in the spleen of the in situ carcinoma model. Mouse spleens were collected, and single-cell suspensions were prepared by mechanical grinding followed by filtration through a 70 μM cell sieve. Lymphocytes were then isolated using Mouse Spleen Lymphocyte Isolation Solution (P8860, Solarbio, Beijing, China). An appropriate amount of cells were resuspended in flow buffer, 5 μl of the anti-mouse primary antibody was added (Table S1), incubated for 15 min at room temperature and protected from light, washed in flow staining buffer and detected by FACS Calibur flow cytometer and Flowjo X was used for Data analysis.

### Western blot

Western blot was utilized to detect the relevant proteins of Nod2/CCN4/NF-κB and TLR4/MYD88 and CDH1/β-catenin signaling pathways. Protein lysates were extracted from the supernatant by lysing cells on ice for 30 min using radioimmunoprecipitation assay (RIPA) lysis buffer containing 1% protease inhibition and 1% protein phosphatase inhibitor, followed by centrifugation at 12,000 × *g* for 15 min. Total protein (20 μg) was separated by electrophoresis in 8% to 12.5% sodium dodecyl sulphate–polyacrylamide gel electrophoresis (SDS-PAGE) and then electrotransferred from the gel onto a 0.45 μM polyvinylidene fluoride membrane. The membranes were blocked with TBST containing 5% skimmed milk for 1 h at 25 °C, incubated with the appropriate proportion of primary antibody (Table S2) overnight at 4 °C, washed three times with TBST, incubated with horseradish peroxidase conjugated secondary antibody of the corresponding species for 1 h at 25 °C, and then washed three times with TBST. The secondary antibodies were conjugated with Chemistar™ High-sig ECL western blot substrate (Tanon, Shanghai) and imaged using Chemi Dox XRS chemiluminescence imaging system (Bio-Rad). ImageJ (NIH, USA) was used to analyze protein bands, and the relative expression of the target proteins was calculated using GAPDH as an internal control.

### Co-immunoprecipitation

Co-immunoprecipitation was conducted to analyze the interaction between Nod2 and CCN4. Protein lysates were extracted from A549 cells as described in part 2.10. An equal volume of protein lysate was pre-incubated with protein A agarose beads on a rotary shaker at 4 °C for 4 h. Antibodies against the target protein (CCN4, Nod2) or IgG were added and incubated with protein A agarose and gently spun overnight at 4 °C. The eluted protein was re-suspended in 2 × SDS for immunoblot analysis. A portion of the protein extract was used as input control.

### Immunofluorescence

Immunofluorescence was adopted to determine the distribution and localization of the pertinent proteins (p65, Nod2, and CCN4). Cells (3 × 10^5^) were inoculated onto cell crawls in 24-well plates, and after co-culture of *V. parvula* with cells, samples were collected and fixed in methanol at − 20 °C for 15 min. Samples were blocked with blocking buffer (1 × PBS/5% goat serum/0.3% Triton X-100) for 1 h at 25 °C and incubated overnight at 4 °C with the addition of murine monoclonal antibody NF-κB p65 (1:100, 66535–1-Ig), followed by incubation with secondary antibody Alexa Fluor 488-labeled goat anti-mouse IgG (A0428, Beyotime, Shanghai) for 1 h at 25 °C. Antifade Mounting Medium with 4′,6-diamidino-2-phenylindole (DAPI) was added to stain cell nuclei. In cell fluorescence co-localization experiments, primary antibodies were added to equal volumes of mouse monoclonal antibody NOD2 (B-4) (1:50, sc-56168) or rabbit polyclonal antibody CCN4 (1:100, 18166–1-AP) and incubated overnight at 4 °C; secondary antibodies were incubated with Alexa Fluor 488-labeled goat anti-mouse IgG and Alexa Fluor 647-labeled goat anti-rat IgG, respectively. The sections were incubated with Alexa Fluor 647-labeled goat anti-rabbit IgG antibody. Photographs were obtained using a fluorescence microscope.

### Identification of the expression pattern, prognostic value, and molecular correlation of CCN4 in LUAD

The difference in the expression level of the CCN4 gene [ENSG00000104415.14] between lung adenocarcinoma (LUAD) tumor samples and adjacent healthy samples were analyzed using public data from The Cancer Genome Atlas (TCGA) database. RNAseq data in TPM format for 598 samples were downloaded and processed using log2(value + 1) transformation. The data were analyzed using the statistical packages in R (version 4.2.1) including stats (version 4.2.1) and car (version 3.1-0) and visualized using ggplot2 package (version 3.3.6). Non-paired sample data analysis was conducted using Wilcoxon rank sum test, while paired sample data analysis was conducted using paired sample t-test method. In addition, the differences in survival probability between the high and low CCN4 expression groups in LUAD were compared using Kaplan–Meier curves. The statistical analysis was performed using the Logrank test, and survival regression was fitted using the survival package (version 3.3.1). The associated p-value obtained by the Logrank test was reported to assess the statistical significance of the results. The results were visualized using the survminer (version 0.4.9) and ggplot2 package (version 3.3.6). The prognosis types were selected to be overall survival (OS) and progress-free interval (PFI). Furthermore, TIMER2 (Tumor Immune Estimation Resource version 2; http://timer.cistrome.org/) (26) was utilized to examine the correlation between the expression levels of CCN4 and Nod2 in LUAD. Specifically, the gene correlation module in TIMER2 was used to calculate the Pearson correlation coefficient and the associated p-value between the two genes in LUAD. The absolute value of the correlation coefficient represents the degree of correlation, with values between 0 and 0.3 indicating weak or no correlation, values between 0.3 and 0.5 indicating weak correlation, values between 0.5 and 0.8 indicating moderate correlation, and values between 0.8 and 1 indicating strong correlation (27).

### Statistical analysis

IBM SPSS 22.0 software and GraphPad Prism 8.0 were used for statistical analyses of the data. The results of measurement data are expressed as mean ± standard deviation. Data conforming to normal distribution and chi-square were tested using the independent sample *t*-test. Data not conforming to normal distribution were tested using the approximate *t*-test. A one-way analysis of variance (ANOVA) was used to compare multiple samples. Statistical significance was established at *p* < 0.05.

## Results

In order to investigate the effects of *V. parvula* on the progression of LUAD, we first used the C57 bl/6j mouse tumor-bearing model and a bacterial cell co-culture model to confirm whether *V. parvula* can promote LUAD proliferation in vivo and in vitro, respectively. Furthermore, we combined transcriptome sequencing and TCGA database to explore the potent molecular mechanism. Finally, we confirmed that Nod2/Ccn4/NF-κB signaling may be the key pathway by which *V. parvula* promotes LUAD.

### *Veillonella* is a critical distinct genus of non-small cell lung cancer

**Fig. 1:**
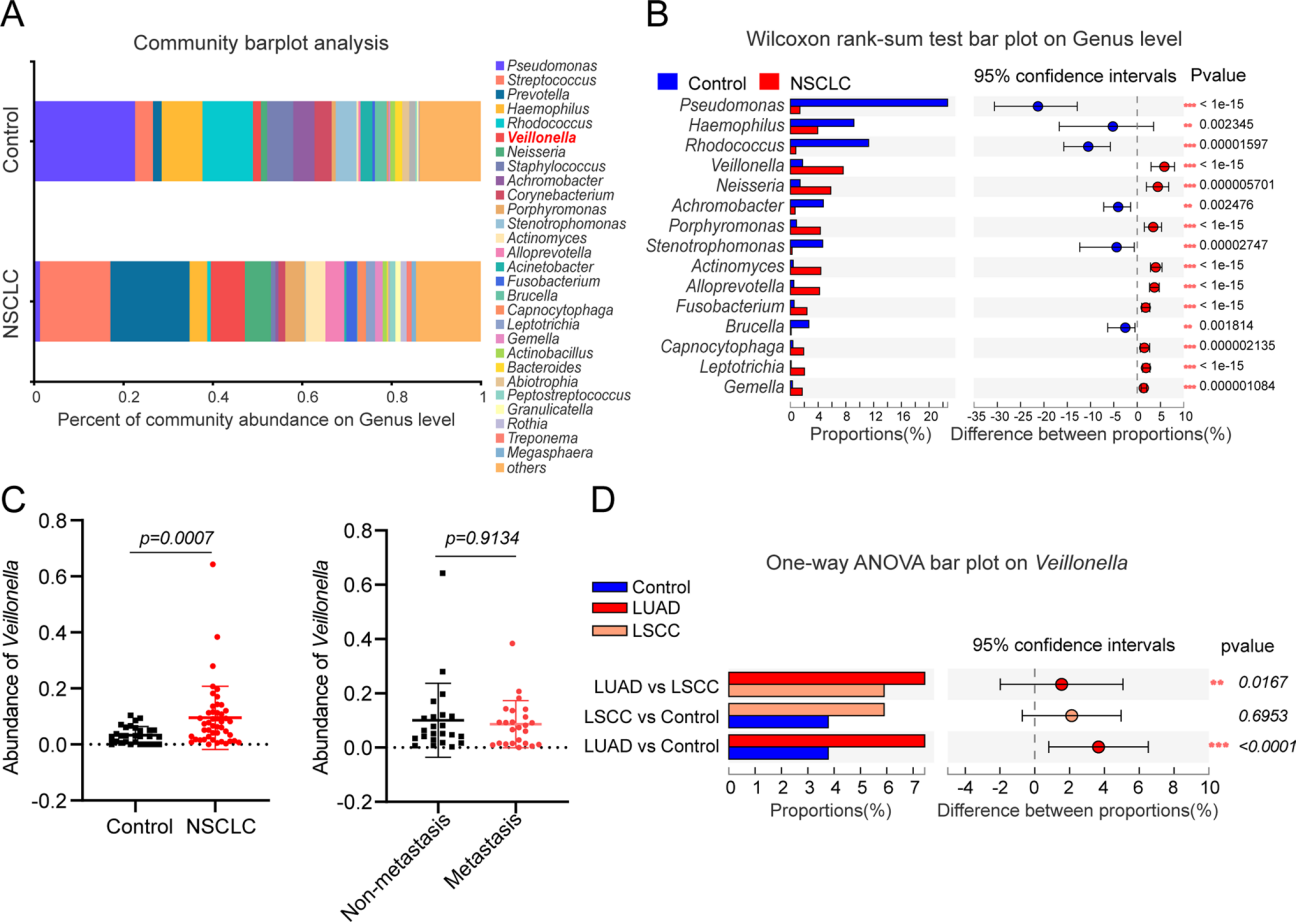
Correlation between *Veillonella parvula* and NSCLC. **A** Bar graph of colony composition; **B** Analysis of significant difference test between groups; **C** Correlation of *V. parvula* abundance with NSCLC and metastasis; **D** Differences in the abundance of *V. parvula* in LUAD and squamous lung cancer, *p < 0.05, **p < 0.01, ***p < 0.001. Control (benign lung disease group, n = 29); non-small cell lung carcinoma (NSCLC, n = 46); non-small cell lung adenocarcinoma (LUAD, n = 25); non-small cell squamous lung cancer (LSCC, n = 21). Metastasis (n = 23) and non-metastasis (n = 23)

Previously, our study reported differences in the microbiota characteristics of alveolar lavage fluid between non-small cell lung cancer (NSCLC) and benign lung diseases (23). Further analysis of the correlation between *Veillonella* abundance and NSCLC indicated that the abundance of the *Veillonella* genus was significantly higher in the NSCLC group compared to that in the control group (accounting for 7.62% of the microbiota in the NSCLC group vs. 1.76% in the control group; *p* < 0.001). However, the high abundance of *Veillonella* did not correlate significantly with the distant metastatic status of NSCLC (Fig. 1A–C). Of the various pathological subtypes, LUAD (*p* < 0.0001) as well as lung squamous carcinoma (*p* < 0.05) were associated with a significantly higher abundance of *Veillonella* than the controls (Fig. 1D). These findings suggest a strong association between *Veillonella* and the progression of LUAD.

### *Veillonella parvula* promotes the progression of lung adenocarcinoma in vivo

**Fig. 2:**
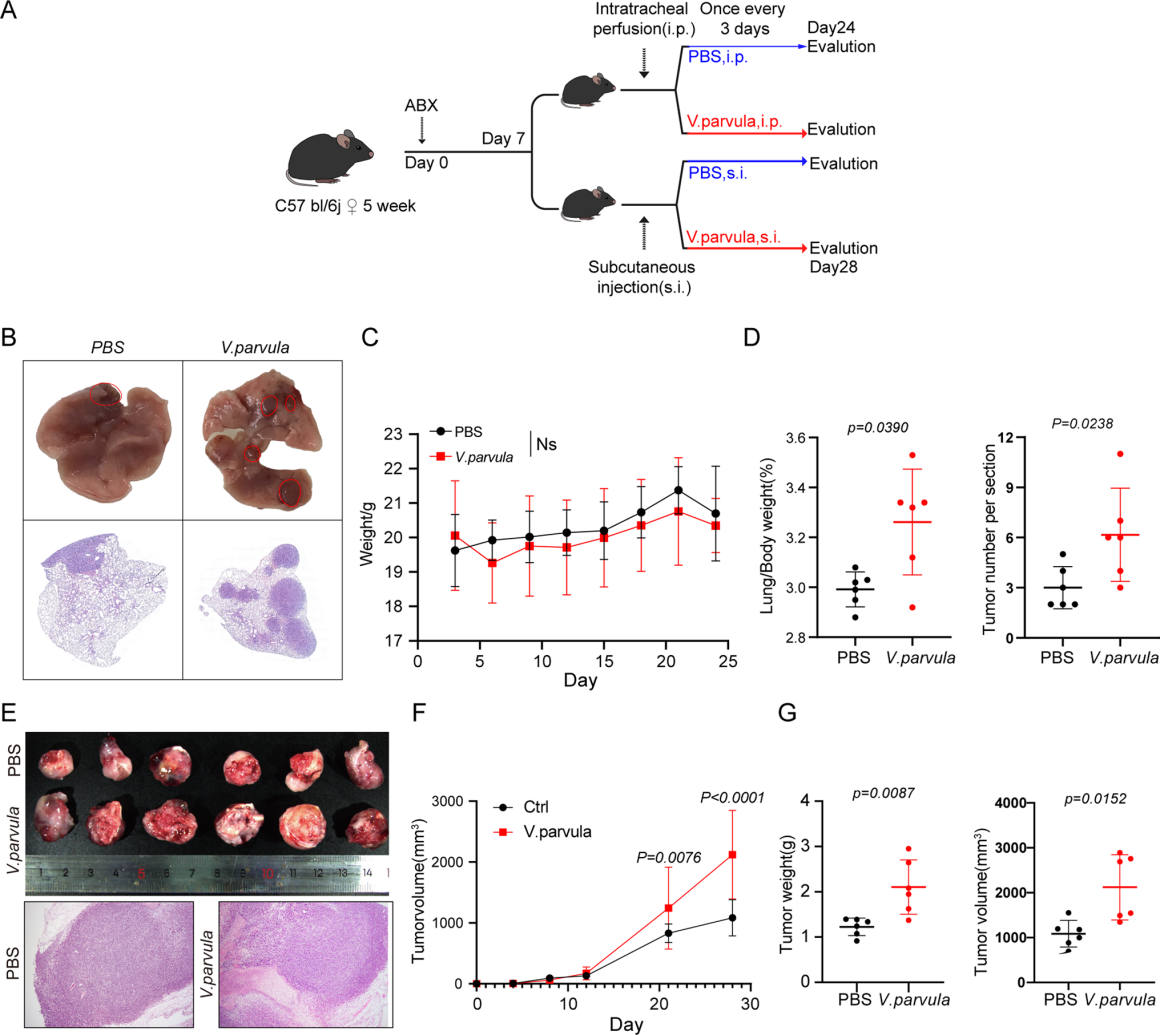
*Veillonella parvula* promotes the progression of lung adenocarcinoma in vivo. **A** Construction of the lung in situ implantation tumor model and the subcutaneous graft tumor model; **B** Representative lung images from C57 bl/6j lung in situ implantation tumor model and H&E staining result; **C** Changes in body weight of in situ cancer mouse models, n = 8 per group; **D** Lung-to-bodyweight ratios and tumor number per analyzed section in two groups; **E** Representative images of LLC tumor from C57 bl/6j subcutaneous graft tumor model and H&E staining result, n = 6 per group; **F** Volume changes of subcutaneous graft tumors in C57 bl/6j mice; **G** Comparison of graft tumor weight and volume, *p* < 0.05, indicates significant difference

To investigate the effect of *V. parvula* on LUAD, the effect of intratracheal instillation and peritumoral injection of *V. parvula* on the development of LLC in mice was observed, with PBS used in the control group (Fig. 2A). Compared to controls, intratracheal instillation of *V. parvula* significantly promoted an increase in tumor burden in situ in the lung, including an increase in the lung-body weight ratio and tumor number and size. However, it did not affect body weight in mice (Fig. 2B–D). In the subcutaneous graft model, peri-tumor injection of *V. parvula* also significantly enhanced the growth of the graft in mice, and this growth-promoting effect occurred at a relatively late stage (Fig. 2E–G). Thus, *V. parvula* promoted the growth of LUAD in vivo.

### Veillonella parvula alters the immune microenvironment in vivo

**Fig. 3:**
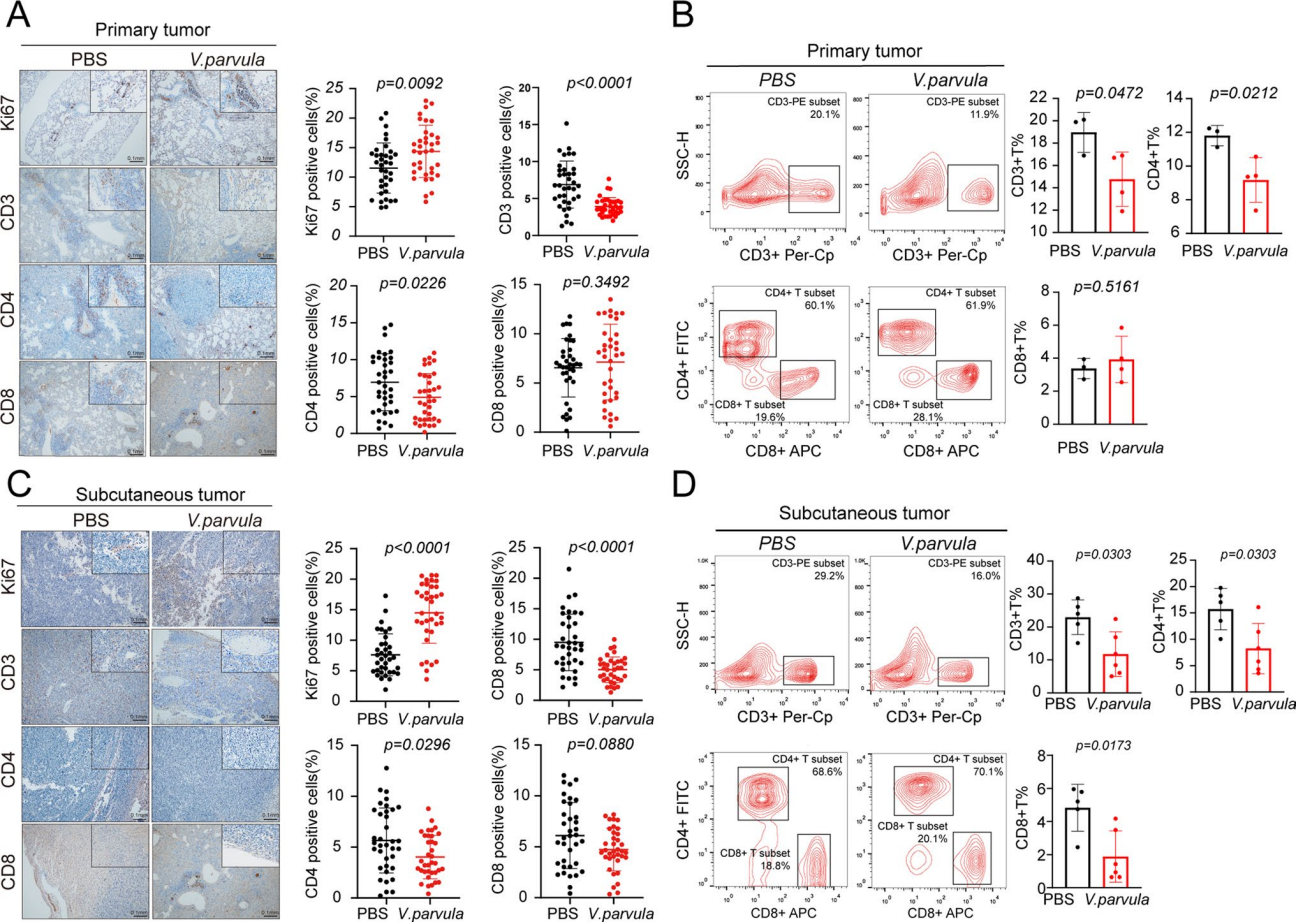
*Veillonella parvula* alters the immune microenvironment in mice. **A** Representative images and quantification of IHC staining for Ki67 and CD3, CD4, CD8 in in situ lung carcinoma C57 bl/6j mice, n = 6 per group, Scale bars, 100 μM; **B** Quantification of indicated CD3^+^, CD4^+^, CD8^+^T cells in the spleen of the in situ carcinoma model, n = 3 to 4 mice per group; **C** Representative images and quantitation of IHC staining for Ki67 and CD3, CD4, CD8 in subcutaneous tumors, n = 6 per group, Scale bars, 100 μM; **D** Quantification of indicated CD3^+^, CD4^+^, CD8^+^ T cells in the spleen of subcutaneous transplant tumor, n = 5 to 6 mice per group. *p* < 0.05 indicates a significant difference

The expression of proliferation index Ki67 was measured by immunohistochemistry, and tumor-infiltrating T lymphocytes and infiltration of splenic T lymphocytes were assessed by flow cytometry. In the in situ carcinoma mouse models, intratracheal perfusion of *V. parvula* promoted Ki67 expression in tumor lesions (*p* = 0.0092) and reduced peripheral and tumor-associated CD3^+^ and CD4^+^ T-lymphocyte infiltration (*p* < 0.05) but did not affect CD8^+^ T-lymphocyte expression (Fig. 3A, B). In subcutaneously transplanted mice, peritumoral injection of *V. parvula* similarly promoted Ki67 expression in the tumor tissue (*p* < 0.0001), reduced peripheral and tumor-associated CD3^+^ and CD4^+^ T-lymphocyte infiltration (*p* < 0.05), and reduced peripheral CD8^+^ T cell infiltration (*p* = 0.0173), but did not affect tumor-infiltrating CD8^+^ T cell expression (*p* = 0.05) (Fig. 3C, D). In summary, *V. parvula* affected tumor infiltration and redistribution of peripheral T lymphocytes, acting as a facilitator of LUAD.

### *Veillonella parvula* promotes the proliferation of lung adenocarcinoma cells in vitro

**Fig. 4:**
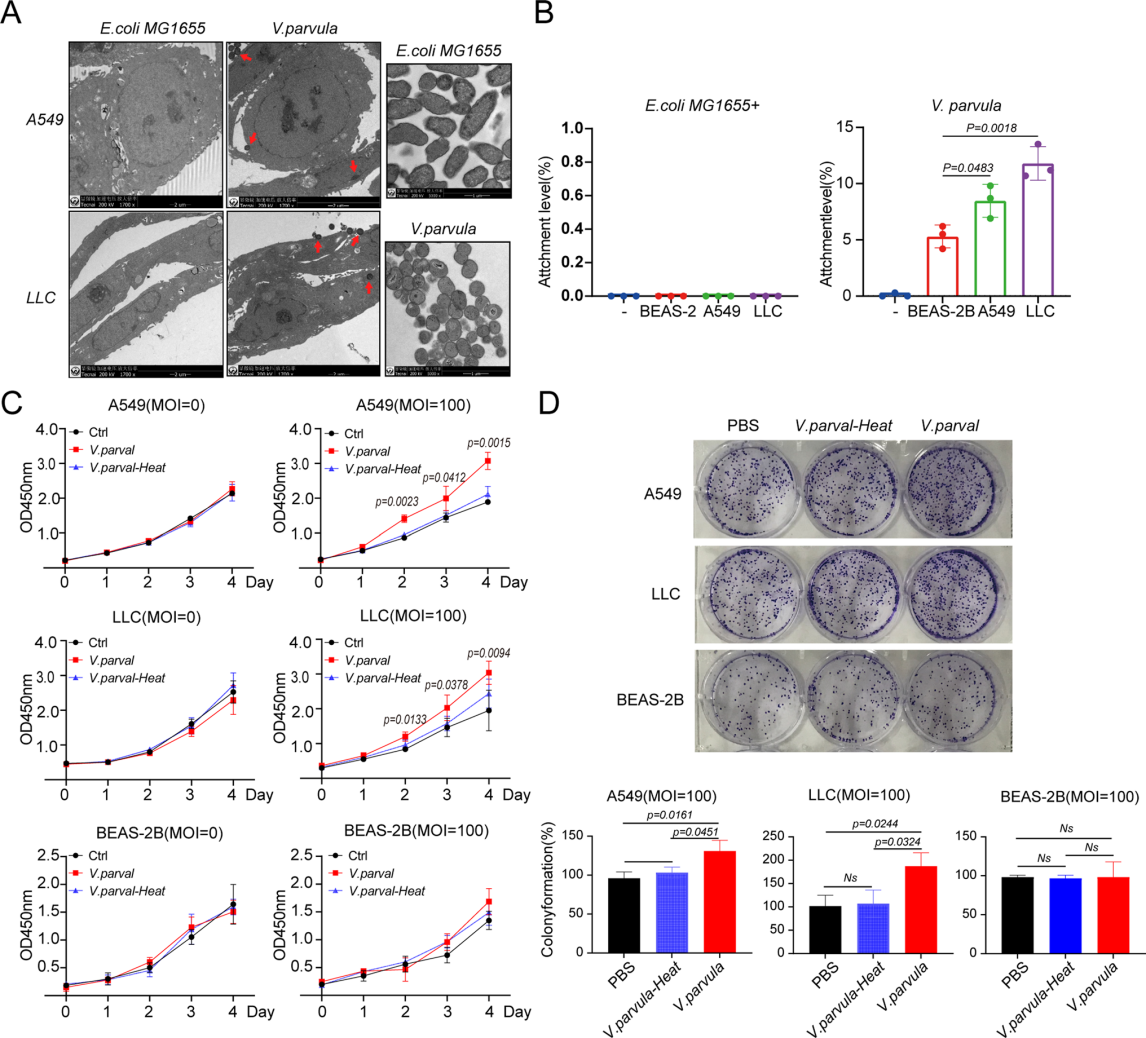
*Veillonella parvula* promotes the proliferation of lung adenocarcinoma cells in vitro. **A** Representative TEM images of *V. parvula* and *E. coli* (MG1655) co-culture with A549 or LLC cells. *E. coli* MG1655 is a negative control. The red arrows indicate *V. parvula*. Scale bars = 1 μM in *V. parvula* and *E. coli* MG1655 (right two panels); 2 μM for the remaining panels; **B** The level of attachment of *V. parvula* or *E. coli* MG1655 on A549, LLC, and BEAS-2B cell lines, MOI = 100 for 1 h; **C** Co-culture *V. parvula* (MOI = 100) or *V. parvula* -Heat (MOI = 100) with A549, LLC, and BEAS-2B cell lines. The proliferation of cells was measured by CCK8 assay; **D** Colony formation of A549, LLC, and BEAS-2B cells co-culture with *V. parvula* or *V. parvula*-Heat at MOI = 100, PBS is the control. *p* < 0.05 indicates significance

A549 and LLC LUAD cell lines and normal lung epithelial BEAS-2B cell line (as control) were used to analyze the growth-promoting effect of *V. parvula* on LUAD in vitro. Electron microscopy revealed that *E. coli* MG1655 was short rod-shaped whereas *V. parvula* was circular. *E. coli* MG1655 did not attach to cells, whereas *V. parvula* could adhere and invade A549 and LLC cells to different degrees (Fig. 4A). The bacterial adhesion assay showed that *V. parvula* exhibited a stronger binding affinity for LUAD cells than BEAS-2B (Fig. 4B). CCK8 and clone formation assays confirmed that active *V. parvula* significantly promoted the proliferation of LUAD cells but did not affect the proliferation of normal BEAS-2B cells; this promoting ability was lost after heat inactivation (Fig. 4C, D). These results suggest that *V. parvula* promotes the proliferation of LUAD cells and is associated with cell adhesion.

### *Veillonella parvula* promotes CCN4 expression in lung adenocarcinoma cells

**Fig. 5:**
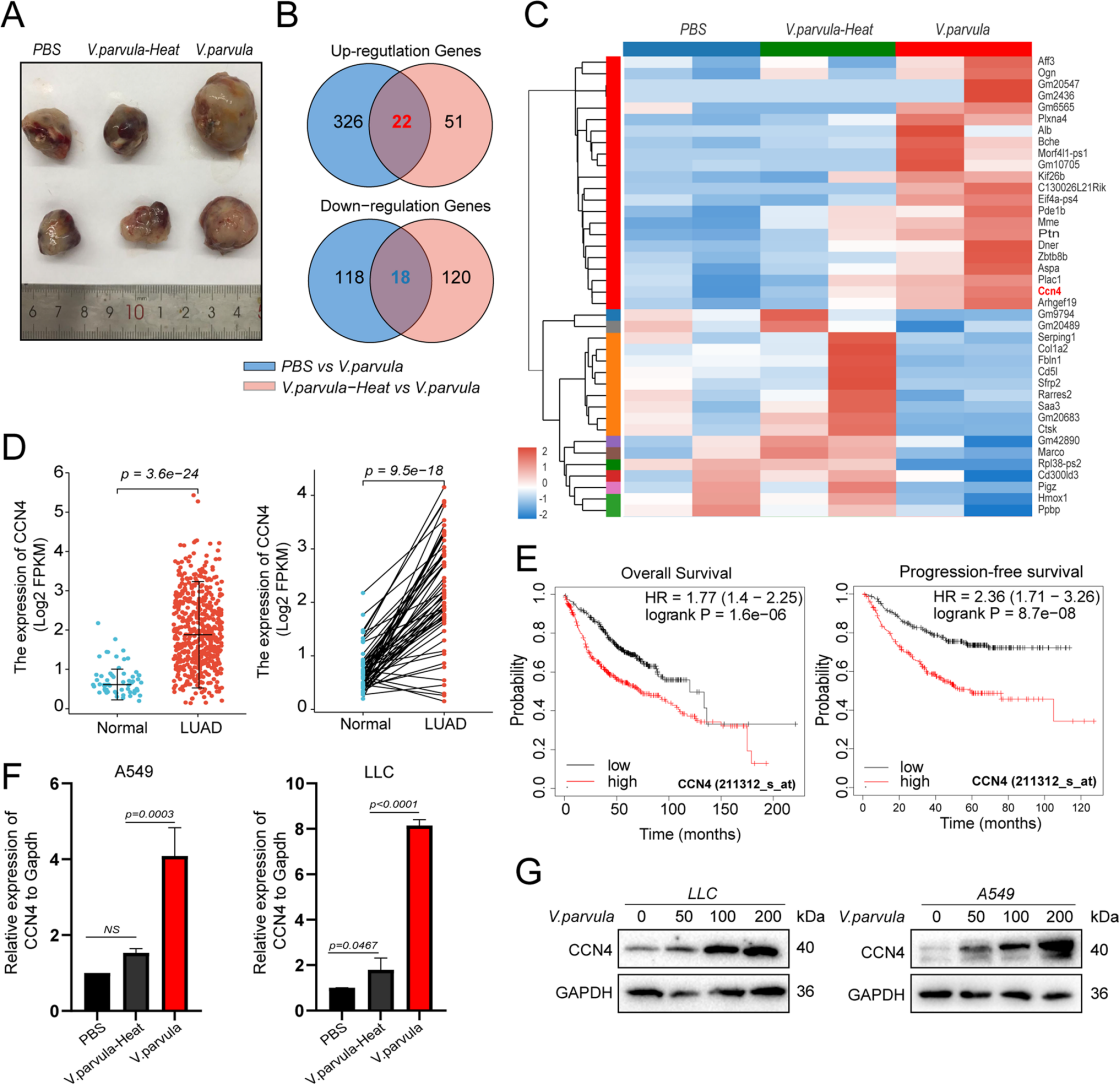
*Veillonella parvula* promotes CCN4 expression in lung adenocarcinoma cells. **A** Effect of PBS, *V. parvula*-Heat, and *V. parvula* on the growth of LLC xenografts, PBS-treated is the control group. **B**-**C** Up/downregulated genes co-regulated between *V. parvula* vs. *V. parvula*-Heat, *V. parvula* vs. PBS, **B** Venn diagram, **C** heatmap; **D** Analysis of CCN4 expression in unpaired (left) and paired (right) LUAD and normal tissue samples based on TCGA data, Normal = 46, LUAD = 521; **E** Correlation of CCN4 expression with OS and PFS in LUAD; **F** Expression of CCN4 mRNA by qRT-PCR; **G** Expression of CCN4 protein by western blotting

To analyze the molecular mechanisms by which *V. parvula* promotes LUAD growth, transcriptome sequencing of *V. parvula*, heat-inactivated *V. parvula*, and PBS-treated transplanted tumors was performed; the results revealed that 22 genes were upregulated, and 18 genes were downregulated after *V. parvula* treatment (Fig. 5A–C). High CCN4 expression in LUAD was associated with poor OS and PFS (Fig. 5D, E, Figure S1, Table S3). The functions of CCN4 are related to integrin binding, cell adhesion, cell–cell signaling, and inflammatory responses (Table S4). Knockdown of CCN4 inhibits the proliferation and migration ability of LLC cells and promotes apoptosis (Figure S2). The qPCR results revealed that *V. parvula* and heat-inactivated *V. parvula* both upregulated CCN4 mRNA expression in LUAD cells, with more significant upregulation by *V. parvula* (Fig. 5F). The immunoblotting results further suggested that *V. parvula* promoted CCN4 protein expression in a dose-dependent manner (Fig. 5G).

### Role of CCN4 in regulating the promotion of lung adenocarcinoma cell proliferation by *V. parvula*

**Fig. 6:**
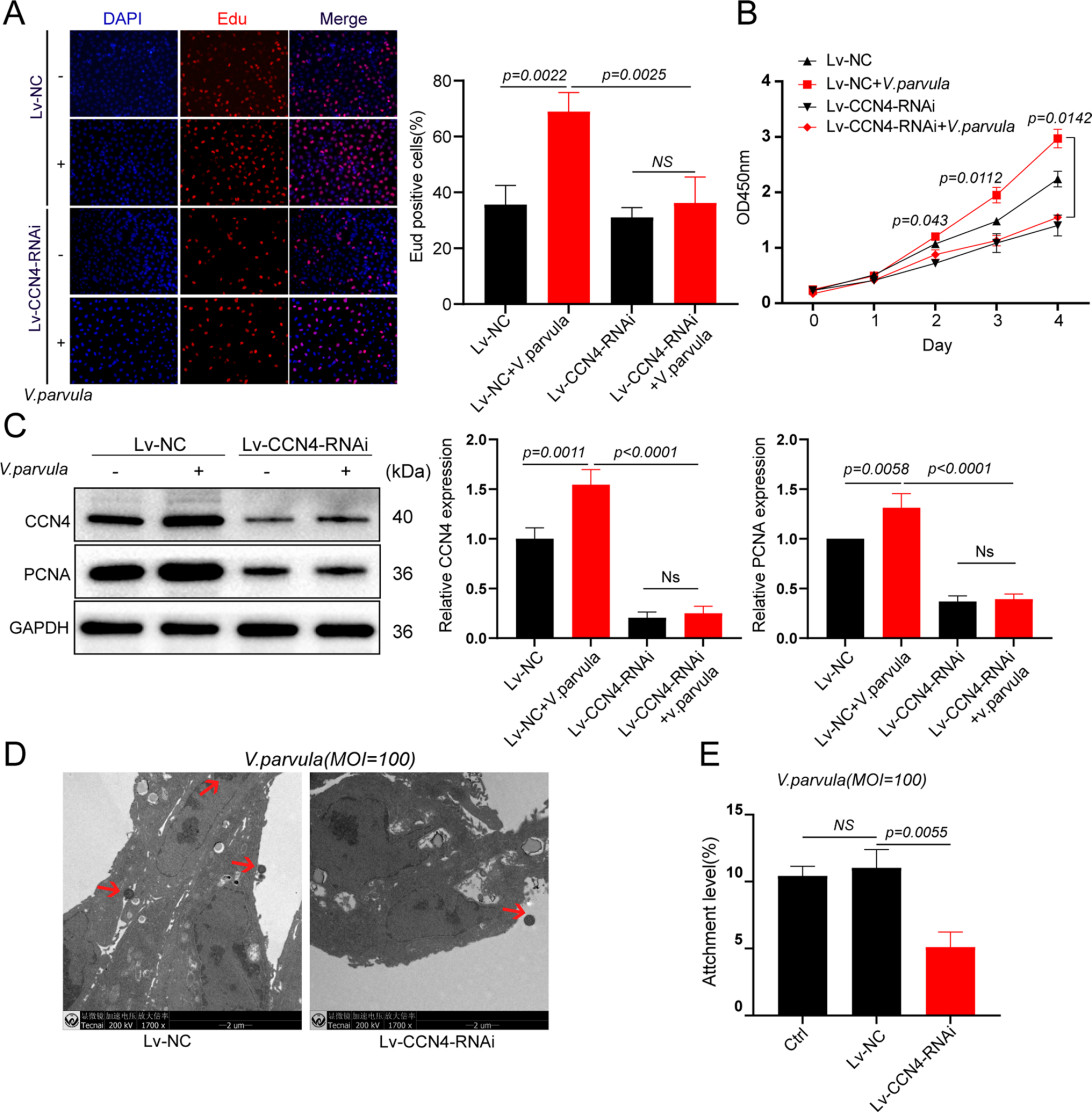
CCN4 involved in regulating the promotion of lung adenocarcinoma cell proliferation by *Veillonella parvula*. **A** EdU cell proliferation assay, DAPI-labelled nuclei (Blue), EdU-labelled cells for DNA replication (Red), Scale Bar: 100 μM; **B** Co-culture *V. parvula* (MOI = 100) with Lv-NC and Lv-CCN4-RNAi from day 0 to 4, respectively, cell proliferation was measured by CCK8 assay; **C** Western blot detection of CCN4, PCNA protein expression; **D** Representative TEM images of *V. parvula* co-culture with Lv-NC, Lv-CCN4-RNAi cells, red arrows indicate *V. paruvula*, scale Bar: 2 μM; **E** Bacterial adhesion assay detects the ability of *V. parvula* to adhere to Lv-NC, Lv-CCN4-RNAi cells. *p* < 0.05 indicate statistical significance

To analyze whether CCN4 regulates the growth-promoting effects of *V. parvula*, LLC cells with knockdown of CCN4 (Lv-CCN4-RNAi) and control cells (Lv-NC) were co-cultured with *V. parvula* at MOI of 100. EdU-positive cells represent cell replication, and the EdU assay revealed that knockdown of CCN4 significantly inhibited the ability of *V. parvula* to promote the proliferation of LLC cells (Fig. 6A). The CCK8 assay also revealed that the knockdown of CCN4 significantly repressed the ability of *V. parvula* to promote proliferation of LLC cells on days 2, 3, and 4 (day 2, *p* = 0.043; day 3, *p* = 0.0112; day 4, *p* = 0.0142) (Fig. 6B). PCNA is an indicator of cell proliferation status, and *V. parvula* was found to promote PCNA expression, whereas knockdown of CCN4 reduced the upregulation of PCNA by *V. parvula* (Fig. 6C). In addition, electron microscopy and bacterial adhesion assays showed that inhibition of CCN4 expression significantly reduced the adhesion level of *V. parvula* to LLC cells (Fig. 6D, E).

### *Veillonella parvula* activates Nod2 and NF-κB signaling pathways in lung adenocarcinoma cells

**Fig. 7:**
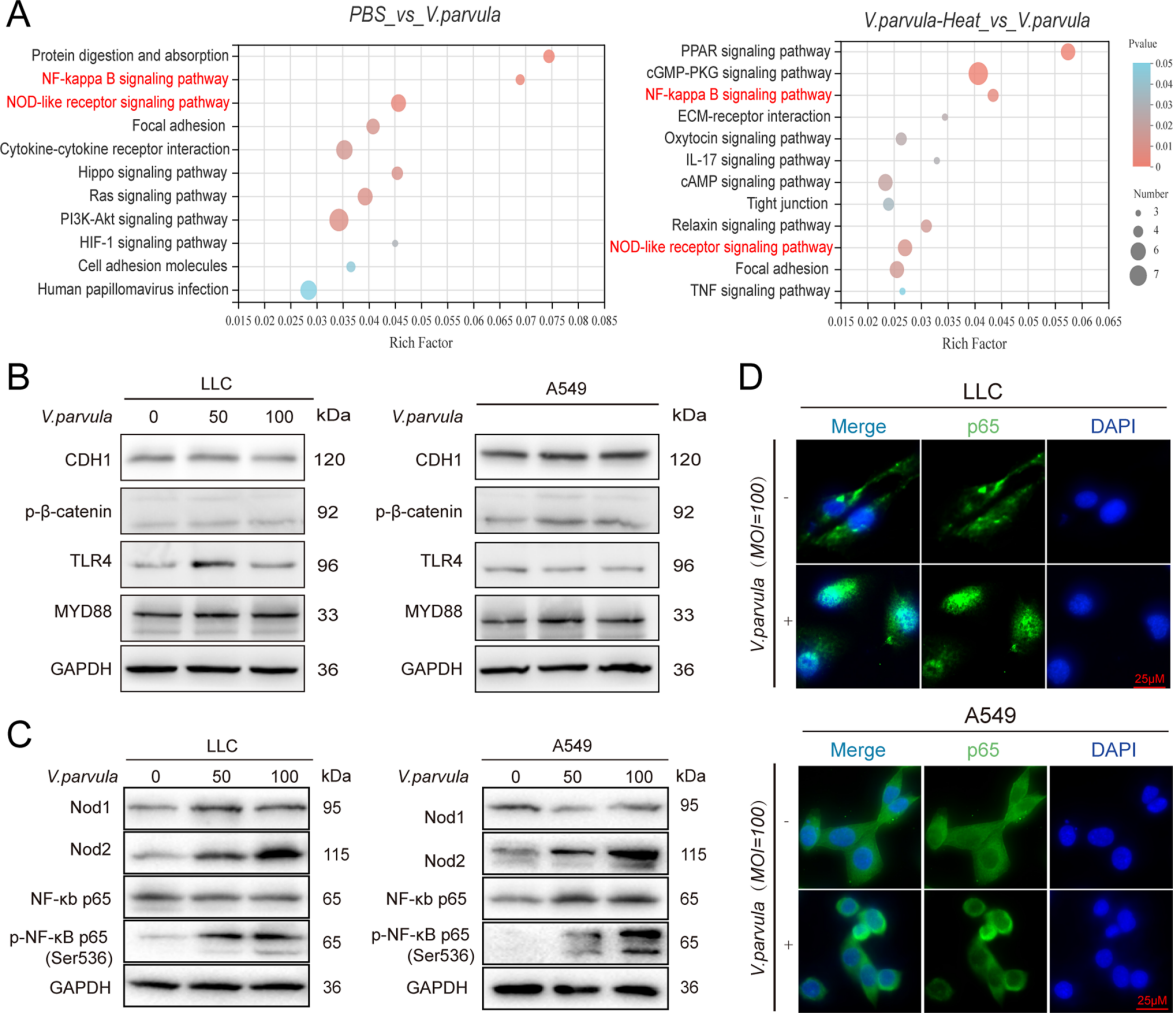
*Veillonella parvula* activates Nod2 and NF-κB signaling pathways. **A** KEGG enrichment analysis of differential genes from transcriptome sequencing; **B** Western blot detects the expression of CDH1, p-β-catenin, TLR4, MYD88, and GAPDH in LLC and A549 cells treated with *V. parvula* at different MOI at 50 and 100; **C** Western blot detects the expression of Nod1, Nod2, NF-κB p65, p-NF-κB p65(Ser536), and GAPDH in LLC and A549 cells treated with *V. parvula* at different MOI at 50 and 100; **D** Immunofluorescence staining assay showed that p65 translocation to the nucleus was significantly increased in LLC and A549 cells treated with *V. parvula*. scale bar, 25 μM

The transcriptome results were analyzed for KEGG enrichment, indicating co-activation of the NOD-like receptor signaling pathway and NF-κB signaling (Fig. 7A). We hypothesized that *V. parvula* promotes LUAD progression by activating the Nod and NF-κB signaling pathways, and we observed that *V. parvula* does not activate TLR4/MYD88 and CDH1/β-catenin signaling pathways in LLC and A549 cells (Fig. 7B). We then measured the expression of NOD-like receptor signaling and NF-κB signaling pathway-related proteins, revealing that *V. parvula* could upregulate Nod2 protein expression in a dose-dependent manner but did not affect Nod1 protein expression (Fig. 7C). In A549 and LLC cells, *V. parvula* promoted increased expression of phosphorylated p65 protein and p65 nuclear translocation, suggesting activation of the NF-κB signaling pathway (Fig. 7C, D). These results suggest that *V. parvula* may mediate the activation of the Nod2/NF-κB signaling pathway, thus promoting the progression of LUAD.

### *Veillonella parvula* activates the NF-κB signaling pathway via Nod2/CCN4 signaling

**Fig. 8:**
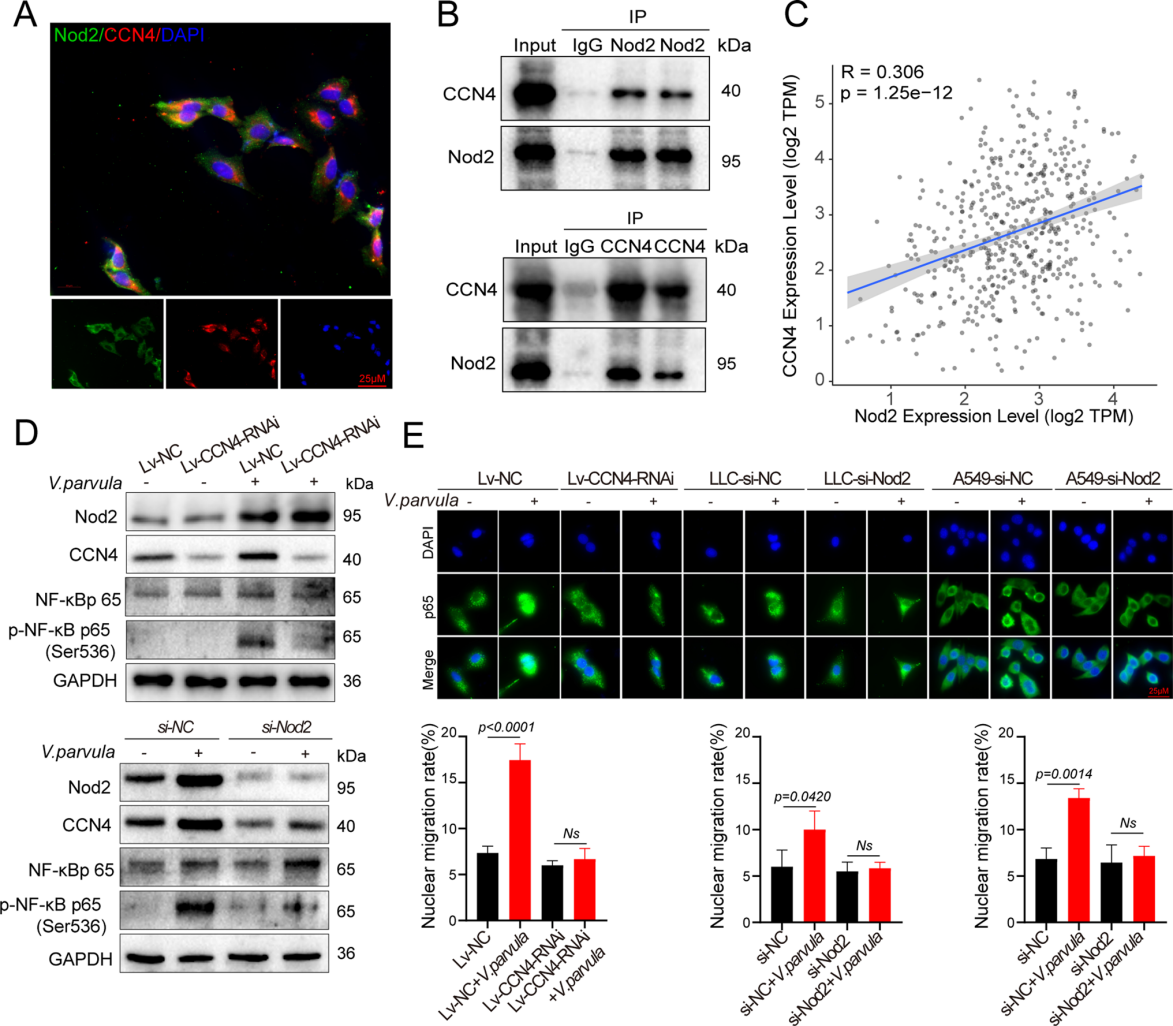
*Veillonella parvula* activates the NF-κB signaling pathway through Nod2/CCN4 signaling. **A** Immunofluorescent analysis of Nod2 and CCN4 localization, green indicates Nod2, red indicates CCN4, and blue indicates the nucleus; **B** Immunoprecipitation analysis of the interaction between Nod2 and CCN4, IgG as control; **C** Correlation of CCN4 with Nod2 expression analyzed by TCGA-LUAD; **D** Western blot analysis of Nod2, CCN4, NF-κB p65, p-NF-κB p65 (Ser536) in cells; **E** Distribution of NF-κB p65 in different cell lines detected by cell immunofluorescence. LLC was transfected with Lv-CCN4-RNAi or Negative vector, and both LLC and A549 were transfected with si-Nod2 and negative vectors

The regulatory association between Nod2 and CCN4 remains unclear. Co-localization of Nod2 with CCN4 was observed by immunofluorescence; both were expressed in the cytoplasm, which suggested an interaction between Nod2 and CCN4 (Fig. 8A, B). Moreover, there was a strong positive correlation between CCN4 and Nod2 expression (R = 0.306, *p* < 0.001). Further analysis revealed that inhibition of CCN4 expression did not alter the induction of Nod2 expression by *V. parvula,* but significantly inhibited the upregulation of CCN4 expression by *V. parvula* when Nod2 was inhibited. Therefore, Nod2 may act upstream of CCN4 (Fig. 8D). Inhibition of CCN4 or Nod2 inhibited *V. parvula*-induced phospho-p65, reduced *V. parvula*-induced p65 nuclear translocation, and inhibited NF-κB pathway activation (Fig. 8E). These findings suggest that *V. parvula* may activate the NF-κB pathway via Nod2/CCN4 signaling to promote LUAD progression.

## Discussion

Increasing evidence suggests that an imbalance in the microbiota is highly associated with the development, progression, and prognosis of cancer. *Veillonella parvula* was enriched in alveolar lavage fluid, tumor tissue, and saliva specimens obtained from patients with lung cancer (28). Similar findings were obtained in our previous study that compared the characteristics of the lung microbiota between benign lung disease and NSCLC (23). *Veillonella parvula* has bn suggested to play an important role in the development and progression of NSCLC; however, the function and mechanism of *V. parvula* in LUAD remain unknown. In this study, we confirmed the ability of *V. parvula* to enhance the growth of LUAD in vitro and explored the molecular mechanisms by which it facilitates the proliferation of LUAD cells.

First, in flora-depleted C57 bl/6j mice, intratracheal perfusion or peritumoral injection of *V. parvula* promoted the growth of LUAD in terms of both tumor number and size. Furthermore, *V. parvula* significantly promoted the proliferative ability of LUAD cells in vitro compared to normal epithelial cells. Adhesion and colonization of bacteria are often initial conditions that influence tumor progression. Bacteria such as *Peptostreptococcus anaerobius*, *Fusobacterium nucleatum*, and *Bacteroides fragilis* promote the progression of colon carcinogenesis after adhesion to the colon epithelium (29–31). Recent studies have also confirmed that *V. parvula* is more likely to adhere to and invade LUAD cells. Therefore, these results provide evidence to explore molecular mechanisms by which *V. parvula* promotes LUAD progression.

The lung microbiota plays an important role in influencing lung immune tolerance; disturbed microbiota impairs immune homeostasis and influences disease progression (32, 33). *F. nucleatum* enrichment can promote the development of intestinal tumors by recruiting tumor-associated macrophages, bone marrow-derived suppressor cells, dendritic cells (DC cells), neutrophils, and other tumor-infiltrating immune cells that play a role in suppressing the T-lymphocyte response (34, 35). Oral administration of probiotic *Bifidobacterium breve* recruits DC and upregulates IL-12 expression to enhance tumor lymphocyte infiltration and exert anti-tumor effects (36). In our study, the intratracheal perfusion of *V. parvula* did not cause serious adverse effects, such as weight loss and blood in stool in mice. Our findings demonstrated that *Veillonella parvula* inhibited the recruitment of tumor-infiltrating T lymphocytes and affected the distribution of CD3^+^ and CD4^+^ T lymphocytes in the peripheral immune environment, with a difference in the effects of different treatments on CD8^+^ T cells, probably influenced by the smaller number of mice. A previous study (37) indicated that B. caecimuris and V. parvula were specifically enriched in non-alcoholic fatty liver disease-hepatocellular carcinoma (NAFLD-HCC), and the significantly enriched microbiota in NAFLD-HCC also included Bacteroides xylanisolvens, Ruminococcus gnavus and Clostridium bolteae. In vitro studies have shown that the microbiota of NAFLD-HCC regulated peripheral immune responses and induced an immunosuppressive phenotype of T cells, characterized by the expansion of regulatory T cells and reduction of CD8 + T cells. And our findings revealed that peritumoral injection of V. parvula reduced peripheral CD8 + T cell infiltration in subcutaneously transplanted mice. Moreover, *V. parvula* enrichment may affect the peripheral and tumor immune microenvironment and thus be involved in the promotion of tumor growth.

Adhesion and invasion are often important mechanisms by which bacteria act on tumors. *Peptostreptococcus anaerobius* induces adhesion to colonic epithelial cells by binding membrane protein PCWBR2 to integrin α2/β1, which induces downstream PI3K/NF-κB signaling cascade activation to promote colon cancer growth (29). Han et al. (30) showed that *F. nucleatum* activates β-catenin signaling by binding to cellular E-cad proteins via the adhesion element, secreting an adhesin known as FadA. No direct effects of *V. parvula* on the proliferation of LUAD cells have been reported. We observed that *V. parvula* has a higher affinity for LUAD cells and can adhere to and invade cells, which may be one of the important ways it affects LUAD growth. Heat inactivation is a conventional method for sterilization of bacterial cultures and is used to study the effects of killed bacteria on host cells. Heat inactivation can affect the surface properties and structures of bacterial cells, which can lead to changes in their adhesion ability to host cells (38). It has been reported that heat inactivation of Escherichia coli and Staphylococcus aureus caused a significant decrease in its adhesive properties (39). E.coli and S.aureus were able to adhere more strongly to surfaces after being exposed to low-temperature inactivation compared to high-temperature inactivation (39). *Veillonella parvula* promotes proliferation and clone formation in LUAD cells, which is lost in heat-inactivated *V. parvula*, indicating that bacteria may exert related effects through components, such as proteins or lipids, worthy of further in-depth study.

**Fig. 9:**
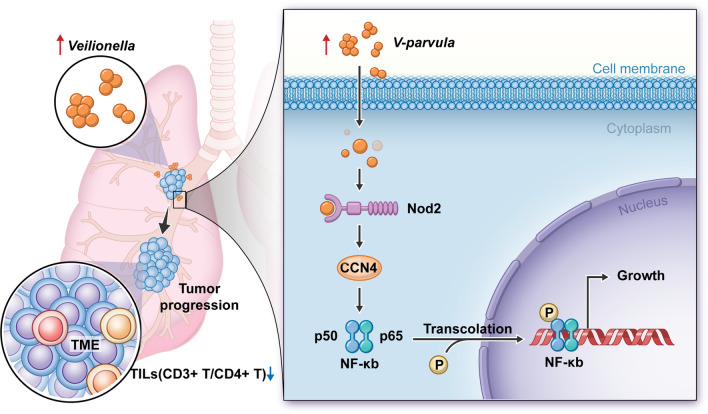
Diagram of *Veillonella parvula* promoting lung adenocarcinoma progression. *V. parvula* is enriched in lung adenocarcinoma and promotes lung adenocarcinoma progression in vivo by inhibiting T-lymphocyte infiltration and in vitro by adhesion to invade lung adenocarcinoma cells via Nod2/CCN4 signalling to activate the NF-κB pathway

CCN4 is involved in regulating cell differentiation, migration, proliferation, and cell adhesion (40) and has been associated with breast cancer (41), colon cancer (42), larynx cancer (43), and malignant melanoma (44). We also confirmed that CCN4 acts as an oncogene for LUAD and is involved in regulating the adhesion and proliferation of LUAD cells by *V. parvula*, which has not been reported previously. This may be related to the engagement of CCN4 in binding to the integrin system, which can confer bacteria with the ability to adhere to and invade epithelial cells and is an important pathway for bacterial cell invasion (45). The activation of the NOD-like and NF-κB signaling pathways may be essential for *V. parvula* to function. NOD-like receptors (NLRs) and Toll-like receptors (TLRs) are the prominent families of innate immune receptors in humans; TLRs primarily bind exogenous substances, and NLRs are involved in endogenous by-products associated with intracellular pathogens and tissue damage (46). In addition, CDH1/Wnt/β-catenin signaling is involved in regulating bacterial effects on host cells (30, 47). Nod1 and Nod2 are the most common NLRs recognized by bacterial cell wall derivatives, bacterial toxins, and viruses (48–50). These are intracellular pattern recognition proteins involved in activating the NF-κB pathway (51, 52). Interestingly, our study found that *V. parvula* affects only Nod2 expression, mediating activation of NF-κB pathway signaling, which induces the expression of pro-inflammatory cytokines, chemokines, and adhesion molecules, to subsequently influence cancer progression (53). In LUAD, Nod2 is correlated with CCN4 expression, and the two proteins colocalize and interact. Furthermore, Nod2 acts as an upstream signal of NF-κB. Therefore, we hypothesized that Nod2/CCN4/NF-κB signaling pathway is an important mechanism for *V. parvula* to regulate LUAD proliferation (Fig. 9).

The current study has some limitations. It did not provide direct evidence of *V. parvula* enrichment in lung cancer tissue samples. In addition, it did not clarify the molecular mechanism of the effect of *V. parvula* in mice and the specific components involved in its action. These scientific questions will be addressed in future studies. Nonetheless, our findings provide an important basis for the prognosis and treatment of LUAD, and they indicate that Nod2 could be a potential therapeutic target for cancer.

## Conclusion

Our study shows that *V. parvula* is closely associated with non-small cell lung adenocarcinoma. Mechanistically, it mediates the activation of the NOD2/CCN4/NF-κB signaling pathway to promote LUAD progression. Thus, our study also provides a potential target for diagnosing and treating LUAD.

## Supplementary Information

Additional file1 (DOCX 838 KB)

## Data Availability

The original contributions presented in the study are included in the article/Additional file. Further inquiries can be directed to the corresponding author.

## Abbreviations

NSCLC: Non-small cell lung cancer
*V. Parvula*: *Veillonella parvula*
TCGA: The Cancer Genome Atlas
KEGG: Kyoto Encyclopedia of Genes and Genomes
CCN4: Cellular Communication Network Factor 4
NOD2: Nucleotide-binding oligomerization domain-containing protein 2
NF-κB: Nuclear Factor kappa B
LLC: Mouse Lewis lung carcinoma cells
LUAD: Lung adenocarcinoma
TSA: Tryptic soy agar
TSB: Tryptic soy broth
MOI: Multiplicity of infection
SDS-PAGE: Sodium dodecyl sulphate–polyacrylamide gel electrophoresis
GAPDH: Glyceraldehyde-3-phosphate dehydrogenase
DAPI: 4′,6-Diamidino-2-phenylindole
DC: Dendritic cells
NAFLD-HCC: Non-alcoholic fatty liver disease-hepatocellular carcinoma
IHC: Immunohistochemistry
