# Supplementary material for: Veillonella parvula promotes the proliferation of lung adenocarcinoma through the nucleotide oligomerization domain 2/cellular communication network factor 4/nuclear factor kappa B pathway
Source: Discov Oncol. 2023 Jul 14;14:129. doi: 10.1007/s12672-023-00748-6 (PMC10349017; doi:10.1007/s12672-023-00748-6)
Supplement: Supplementary file 1 — Additional file1 (DOCX 838 KB) [file 12672_2023_748_MOESM1_ESM.docx]

**Supplementary materials and methods**

**Histology**

Mouse lung and tumor tissues were estimated with 4% paraformaldehyde and sectioned after paraffin embedding. Slides were dewaxed and stained with hematoxylin-eosin (H&E), dehydrated in ethanol and transparent in xylene, and sealed after Lung section was scanned using Tissue FAXS (Tissue Gnostics) and analyzed using the HistoQuest software (For IHC, paraffin sections were dewaxed and stained by standard immunohistochemical procedures, incubated overnight at 4°C with primary antibody, incubated with secondary antibody, DAB stained, sealed with neutral resin and scanned for photographs. described.

**CCK8 test**

BEAS-2B, A549 and LLC were treated separately: control (PBS), *V.parvula*-treated group, *V.parvula-Heat*: heat-inactivated *V.parvula* was prepared by placing in 90°C for 20 min. adjusting the cell concentration to 5×10^4/^ml, taking 100μl uniformly inoculated in 96-well plates, 3 replicate wells per group, placed in incubate at 37℃ 5% incubator for 12h until the cells are plastered, replace the fresh medium containing *V.parvula*, *V.parvula*-Heat with MOI=100 and PBS as control. The cells were incubated under anaerobic conditions for 12h, then transferred to a 37°C 5% cell culture incubator for 4 days. 10μl/well of CCK8 reagent was added at the corresponding time and incubated for 2h at 37°C. OD450nm values were determined using a Varioskan™ flash multimode reader (Thermo Fisher Scientific, Waltham, MA, USA) to determine OD450nm values.

**Clone formation assay**

Take logarithmic growth phase cells, adjust the cell density to 2×103 cell/well inoculated into 6-well plates, experimental grouping and cell and bacteria co-culture method as CCK8 test, incubated at 37℃ 5% CO2 and saturated humidity in the cell culture incubator for 2-3 weeks, see the cell cloning point >50 cells, discard the supernatant, add 4% paraformaldehyde fixed cells 5mL fixed for 15 minutes. Then remove the fixation solution, add appropriate amount of 0.1% crystalline violet for staining solution for 15 min and take pictures for counting.

**qRT-PCR**

After inoculation of 2×105 cells/well of LLC in a 6-well plate, the cells were plastered for 12 h. MOI=100 *V.parvula* was added and co-cultured under anaerobic conditions for 12 h. After that, the cells were transferred to normal culture conditions and continued to be cultured until they reached 80% confluence. The RNA concentration was measured by ultra-micro-UV spectrophotometer (Thermo Scientific, Massachusetts, USA), and the RNA was reverse transcribed into cDNA using an Evo M-MLV RT Premix for qPCR (AG11706, Accurate Biology, Hunan, China). Subsequently, the RNA was reverse transcribed into cDNA using the SYBR® Green Premix Pro Taq HS qPCR Kit (AG11701, Accurate Biology, Hunan, China) and the CFX connetTM fluorescent quantitative PCR detection system (Bio-Rad). The cDNA was amplified using the mouse Ccn4 forward primer (5'-AACTGCATAGCCTACACTAGTC-3') and reverse primer (5'-AACTGCATAGCCTACACTAGTC-3'). Primer(5'-ATTGACGTTAGAGATCCGAGTG-3') were purchased from Shanghai Sangon Biological Engineering Co., Ltd. The relative quantification of the genes was determined according to the 2-ΔΔCt method. GAPDH was used as an endogenous control and each sample was analysed three times.

**Table S1. Primary antibody for FCM**

| Target | Source | Cat |
| --- | --- | --- |
| Anti-Mouse CD8α, APC | Multi-Science | Clone:53-6.7 |
| Anti-Mouse CD3ε-PerCP-Cy5.5 | Multi-Science | Clone:145-2C11 |
| Anti-Mouse CD4, FITC | Multi-Science | Clone: GK1.5 |

**Table S2. Primary antibody for IHC, Western blot and IF**

| Target | Source | Cat | Dilution |  |
| --- | --- | --- | --- | --- |
| Ki67 | Cell Signaling Technology | #12202 | 1:200 | IHC |
| CD3 | Proteintech | 60181-1-lg | 1:100 | IHC |
| CD4 | Cell Signaling Technology | #25229 | 1:100 | IHC |
| CD8 | Proteintech | 10494-1-AP | 1:200 | IHC |
| CCN4 | Proteintech | 18166-1-AP | 1:1000/1:100 | WB, IF |
| NF-κB p65 | Proteintech | 66535-1-Ig | 1:3000/1:100 | WB, IF |
| CDH1 | Proteintech | 20874-1-AP | 1:3000 | WB |
| GPADH | Proteintech | 10494-1-AP | 1:5000 | WB |
| TLR4 | Proteintech | 19811-1-AP | 1:1500 | WB |
| MYD88 | Proteintech | 23230-1-AP | 1:4000 | WB |
| PCNA | Proteintech | 10205-2-Ap | 1:5000 | WB |
| Phospho-NF-κB p65 (Ser536) | Cell Signaling Technology | #3033 | 1:1000 | WB |
| Phospho-β-Catenin (Ser675) | Cell Signaling Technology | # 4176 | 1:1000 | WB |
| Nod1 (B-4) | Santa Cruz Biotechnology, | sc-398696 | 1:2000 | WB |
| Nod2 (B-4) | Santa Cruz Biotechnology | sc-56168 | 1:2000/1:100/1:50 | WB/IF/IP |
| CCN4(WISP1-A-9) | Santa Cruz Biotechnology | sc-133126 | 1:50 | IP |
| HRP-labeled Goat Anti-Rabbit IgG(H+L) | Beyotime | A0208 | 1:1000 | WB |
| HRP-labeled Goat Anti-Mouse IgG(H+L) | Beyotime | A0216 | 1:1000 | WB |
| Alexa Fluor 488-labeled Goat Anti-Mouse IgG(H+L) | Beyotime | A0428 | 1:100 | IF |
| Alexa Fluor 647-labeled Goat Anti-Rabbit IgG(H+L) | Beyotime | A0468 | 1:100 | IF |

**Table S3.** Correlation of differential genes with overall survival prognosis in lung adenocarcinoma based on Kaplan-Meier analysis

|  | Low expression cohort (months) | High expression cohort (months) | HR | logrank P |
| --- | --- | --- | --- | --- |
| Up-genes |  |  |  |  |
| KIF26B | 107 | 108.97 | 1.13(0.88-1.43) | 0.34 |
| DNER | 96.2 | 127 | 0.8(0.63-1.02) | 0.073 |
| PLAC1 | 125.77 | 73.3 | 1.71(1.35-2.16) | 7.10E-16 |
| CCN4 | 125.77 | 69.93 | 1.7(1.34-2.15) | 7.90E-06 |
| ARHGEF19 | 95.07 | 110.27 | 0.94(0.74-1.19) | 0.59 |
| ZBTB8B | 108.97 | 103 | 0.9(0.7-1.14) | 0.37 |
| Down-genes |  |  |  |  |
| MARCO | 99 | 107 | 0.98(0.78*1.24) | 0.88 |
| PIGZ | 117.33 | 88.7 | 1.23(0.98-1.56) | 0.079 |
| HMOX1 | 127 | 57 | 2.28(1.78-2.9) | 1.00E-11 |
| PPBP | 107 | 96.2 | 1.29(1.02-1.62) | 0.034 |
| SERPING1 | 108.97 | 96.2 | 1.1(0.87-1.39) | 0.42 |
| FBLN1 | 90 | 127 | 0.77(0.61-0.97) | 0.029 |
| CD5L | 108.97 | 76 | 1.38(1.09-1.74) | 0.0071 |
| RARRES2 | 119.87 | 79.87 | 1.4(1.11-1.77) | 0.0042 |

**Table S4.**Gene ontology annotations

|  | FBLN1 | CCN4 | PLAC1 |
| --- | --- | --- | --- |
| Molecular function | - calcium ion binding | - heparin-binding |  |
|  | - extracellular matrix structural constituent | - insulin-like growth factor binding |  |
|  | - fibrinogen binding | - integrin binding |  |
|  | - fibronectin binding |  |  |
|  | - identical protein binding |  |  |
|  | - integrin binding |  |  |
|  | - peptidase activator activity |  |  |
|  | - protein-containing complex binding |  |  |
|  | - protein C-terminus binding |  |  |
| Biological process | - blood coagulation, fibrin clot formation | - bone development | - placenta development |
|  | - embryo implantation | - cell adhesion |  |
|  | - extracellular matrix organization | - cell-cell signaling |  |
|  | - negative regulation of cell adhesion | - glucose homeostasis |  |
|  | - negative regulation of cell motility | - negative regulation of cell death |  |
|  | - negative regulation of ERK1 and ERK2 cascade | - negative regulation of chondrocyte differentiation |  |
|  | - negative regulation of protein phosphorylation | - regulation of cytokine production |  |
|  | - negative regulation of stem cell proliferation | - signal transduction |  |
|  | - negative regulation of substrate adhesion-dependent cell spreading | - osteoclast differentiation |  |
|  | - negative regulation of transformation of host cell by a virus | - positive regulation of inflammatory response |  |
|  | - negative regulation of transforming growth factor beta production | - positive regulation of wound healing |  |
|  | - positive regulation of fibroblast proliferation | - positive regulation of smooth muscle cell migration |  |
|  | - positive regulation of gene expression | - positive regulation of smooth muscle cell proliferation |  |
|  | - positive regulation of substrate-dependent cell migration, cell attachment to substrate | - positive regulation of Wnt signaling pathway |  |


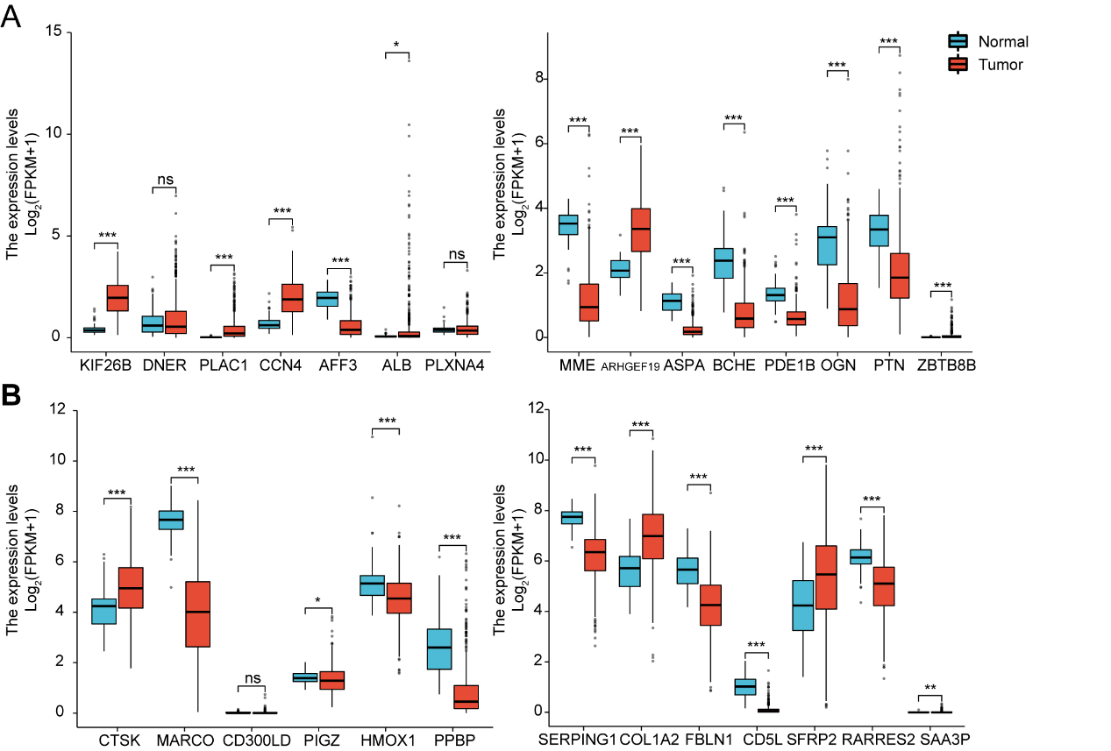


Figure S1. Differential gene expression in lung adenocarcinoma and normal tissues based on TCGA analysis. A. Up-regulation Genes; B. Down-regulation Genes,


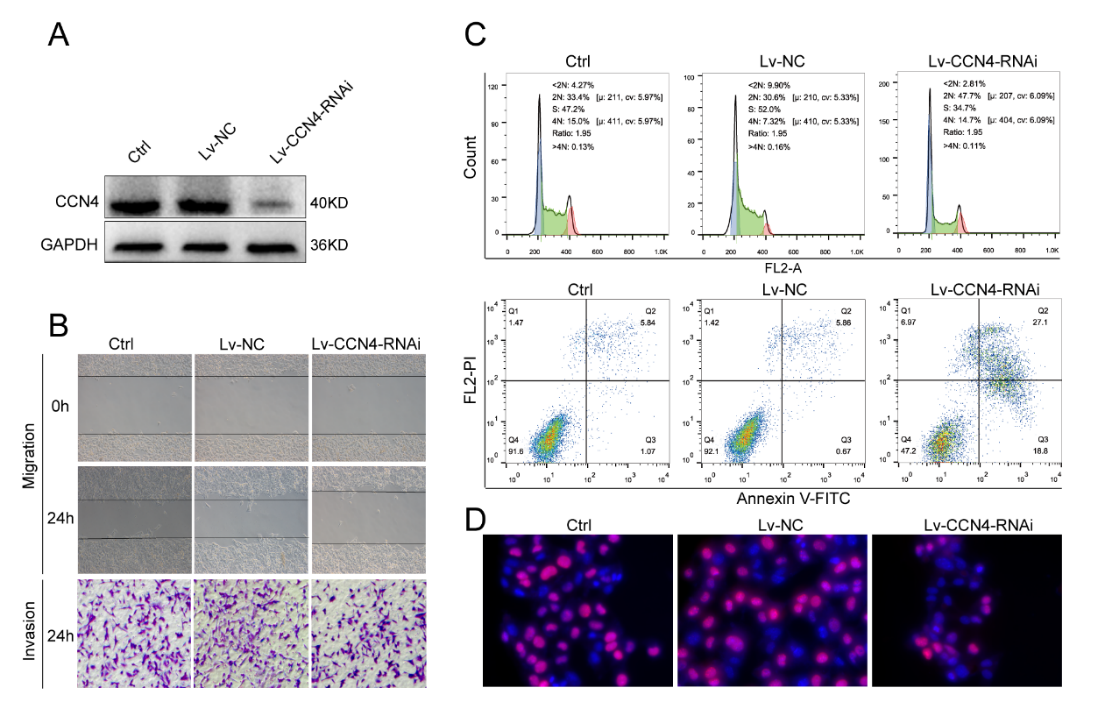


Figure S2 CCN4 knockdown impair the proliferation, migration of LLC cell line. A. Western blot detect expression of CCN4; B. Wound healing assay and migration assay; C. Cell cycle and apoptosis assays；D. Edu assay
